# Effect of selected gastrointestinal parasites and viral agents on fecal S100A12 concentrations in puppies as a potential comparative model

**DOI:** 10.1186/s13071-018-2841-5

**Published:** 2018-04-17

**Authors:** Romy M. Heilmann, Aurélien Grellet, Niels Grützner, Shannon M. Cranford, Jan S. Suchodolski, Sylvie Chastant-Maillard, Jörg M. Steiner

**Affiliations:** 1 Small Animal Clinic, College of Veterinary Medicine, University of Leipzig, An den Tierkliniken 23, DE-04103 Leipzig, Germany; 2 Gastrointestinal Laboratory, Department of Small Animal Clinical Sciences, College of Veterinary Medicine and Biomedical Sciences, Texas A&M University, 465 Raymond Stotzer Pkwy, College Station, TX 77843-4474 USA; 3 NeoCare, IHAP, University of Toulouse, INRA, ENVT, 23 chemin des Capelles, BP 87614, 31076 Toulouse Cedex 03, France; 4 Institute of Agricultural and Nutritional Sciences, Martin Luther University Halle-Wittenberg, Theodor-Lieser St 11, DE-06120 Halle (Saale), Germany

**Keywords:** Biomarker, Canine, Calgranulin C, Diarrhea, Enteropathogen, Parasite, Virus

## Abstract

**Background:**

Previous data suggest that fecal S100A12 has clinical utility as a biomarker of chronic gastrointestinal inflammation (idiopathic inflammatory bowel disease) in both people and dogs, but the effect of gastrointestinal pathogens on fecal S100A12 concentrations is largely unknown. The role of S100A12 in parasite and viral infections is also difficult to study in traditional animal models due to the lack of S100A12 expression in rodents. Thus, the aim of this study was to evaluate fecal S100A12 concentrations in a cohort of puppies with intestinal parasites (*Cystoisospora* spp., *Toxocara canis*, *Giardia* sp.) and viral agents that are frequently encountered and known to cause gastrointestinal signs in dogs (coronavirus, parvovirus) as a comparative model.

**Methods:**

Spot fecal samples were collected from 307 puppies [median age (range): 7 (4−13) weeks; 29 different breeds] in French breeding kennels, and fecal scores (semiquantitative system; scores 1−13) were assigned. Fecal samples were tested for *Cystoisospora* spp. (*C. canis* and *C. ohioensis*), *Toxocara canis*, *Giardia* sp., as well as canine coronavirus (CCV) and parvovirus (CPV). S100A12 concentrations were measured in all fecal samples using an in-house radioimmunoassay. Statistical analyses were performed using non-parametric 2-group or multiple-group comparisons, non-parametric correlation analysis, association testing between nominal variables, and construction of a multivariate mixed model.

**Results:**

Fecal S100A12 concentrations ranged from < 24−14,363 ng/g. Univariate analysis only showed increased fecal S100A12 concentrations in dogs shedding *Cystoisospora* spp. (*P* = 0.0384) and in dogs infected with parvovirus (*P* = 0.0277), whereas dogs infected with coronavirus had decreased fecal S100A12 concentrations (*P* = 0.0345). However, shedding of any single enteropathogen did not affect fecal S100A12 concentrations in multivariate analysis (all *P* > 0.05) in this study. Only fecal score and breed size had an effect on fecal S100A12 concentrations in multivariate analysis (*P* < 0.0001).

**Conclusions:**

An infection with any single enteropathogen tested in this study is unlikely to alter fecal S100A12 concentrations, and these preliminary data are important for further studies evaluating fecal S100A12 concentrations in dogs or when using fecal S100A12 concentrations as a biomarker in patients with chronic idiopathic gastrointestinal inflammation.

## Background

S100A12, also referred to as calgranulin C, belongs to the calgranulin subfamily of the highly conserved S100 proteins [1, 2]. The S100 superfamily of EF-hand proteins has a high Ca^2+^-binding capacity and binding sites for other divalent cations such as Zn^2+^ and Cu^2+^; oligomerization in the presence of Ca^2+^ and Zn^2+^ has been shown to be essential for the target protein interaction and function of S100A12 [3–5].

Intracellular S100A12 protein is mainly localized to the cytosol, and a number of different target proteins of S100A12 suggest that this protein has regulatory functions [6–8]. Extracellular S100A12 has cytokine-like effects and functions as a phagocyte-specific danger-associated molecule (alarmin), with a central role in immune responses [9]. S100A12 plays a role in host defense against microorganisms and parasites [10] by sequestering essential trace elements such as Cu^2+^ [3], and the protein also has chemotactic properties especially for monocytes and mast cells [6, 11, 12]. S100A12 has also been suggested to counteract tissue damage by competing with matrix metalloproteinases (MMPs) for Zn^2+^-binding [13]. The S100A12 protein is a ligand for the pattern recognition receptor RAGE (receptor for advanced glycation end products) and activates the nuclear factor kappa B (NF-kB) pathway [6, 14], a mechanism linked to the pathogenesis of autoimmune disorders such as inflammatory bowel disease (IBD) in humans [6, 15, 16].

In human medicine, S100A12 has been reported to be a sensitive and specific marker of localized inflammatory disease processes, such as gastrointestinal inflammation, and to be increased in fecal samples from patients with IBD (active Crohn’s disease and ulcerative colitis) [16, 17]. Recent studies suggest that fecal S100A12 concentrations may also be clinically useful as a biomarker of inflammation in dogs [18, 19]. In dogs with chronic idiopathic gastrointestinal inflammation (IBD), fecal canine S100A12 concentrations were increased and correlated with the severity of clinical disease, endoscopic changes observed in the intestine and the severity of histologic lesions in the colon [18]. Higher fecal S100A12 concentrations were also shown to be associated with a negative outcome in dogs with IBD [19]. The role of S100A12 as a biomarker of inflammation is currently an area of intensive research in both human and veterinary medicine, and targeting the RAGE-S100A12 axis may also open new therapeutic avenues.

Only very few studies have been published about the effect of enteric parasite infections [20] or enteropathogenic viruses [17] on fecal S100A12 concentrations in humans, and the role of S100A12 in parasite and viral infections is difficult to study using a traditional rodent animal model due to the lack of S100A12 in rodents [21]. Although the function of S100A12 may be species-specific, the existence of a homologue of this calgranulin protein in dogs [22] appears to render the dog an ideal model for studying S100A12 biology and the RAGE-S100A12 axis in human patients.

To the authors’ knowledge, the possibility of an effect of gastrointestinal pathogens on fecal S100A12 concentrations in dogs has not yet been evaluated and reported, but this information will also be important for any further evaluation of the clinical utility of fecal S100A12 as a biomarker in dogs with IBD. Based on the literature available [10, 17, 20], we hypothesized that infections with enteric parasites - but not enteropathogenic viruses - are associated with increased fecal canine S100A12 concentrations, and that these etiologies need to be ruled out when using fecal S100A12 as a biomarker in dogs with IBD. Thus, the aim of this study was to evaluate the effect of the presence and numbers of selected endoparasites and viral agents that are frequently encountered and known to cause gastrointestinal signs in dogs on fecal canine S100A12 concentrations in a large cohort of puppies. Because biomarkers can be affected by patient physiological factors such as age, sex, or size [23], the effect of these parameters was also evaluated.

## Methods

### Study population and ethics approval

A total of 307 purebred dogs ≤ 13 weeks of age from several (*n* = 33) French breeding kennels (from a total of 67 litters) were included in this study. These dogs were apparently healthy, with the exception that some dogs had an abnormal fecal consistency. Details about the characteristics of these dogs, some of which were included in a previous study, have been published [24]. The breeders’ consent was obtained prior to enrollment of dogs and fecal sample collection.

### Sample collection

A fecal specimen was collected from each dog after natural defecation and was prepared and stored as previously described [24]. Depending on the mean adult body weight of their respective breed, puppies were categorized into small breed dogs (≤ 25 kg average adult body weight) or large breed dogs (> 25 kg average adult weight) [25]. For each puppy, fecal quality was evaluated by a single operator (AG) using a 13-point semiquantitative scale (1–13 from liquid to dry consistency) as previously described [25]. Thresholds for an abnormal fecal consistency were applied dependent on breed size and age: feces with a score of ≤ 5 were considered abnormal for large breed puppies regardless of age, whereas for small breed puppies, fecal scores ≤ 6 and ≤ 7 were classified as abnormal for 4- to 5-week-old puppies and for older puppies, respectively [25]. Following stool collection, a rectal swab was collected from each dog for viral pathogen analysis [24].

### Enteropathogen analyses

Fecal samples were tested for the major enteropathogens causing diarrhea in weanling puppies (*Toxocara canis*, *Cystoisospora* spp., *Giardia* spp., canine coronavirus and canine parvovirus type 2) as previously described [24].

Briefly, an aliquot (approximately 5 g) of each fecal sample was used for routine fecal examination using the standard McMaster flotation technique and a second aliquot (approximately 100 mg feces) was used to quantify *Giardia* spp. antigen (ProSpecT-Giardia Microplate ELISA kit, Remel, France) per gram of wet feces [24].

The fecal material obtained *via* rectal swab was tested for the presence of CCV-RNA and CPV2-DNA by qRT-PCR and qPCR, respectively [24, 25]. Results from duplicate PCR analyses from the extracted DNA (i.e. 2 PCR assay were performed for each fecal extract) were expressed semi-quantitatively as virus loads. Puppies were classified as infected by CPV2 and CCV if viral loads were > 10^10.3^ and > 10^9.3^ copies, respectively [24, 25].

### Fecal S100A12 analysis

Another single-spot fecal aliquot (0.8 ± 0.2 g) from each dog was separated and stored frozen (-20 °C) until shipped to the Gastrointestinal Laboratory at Texas A&M University, where this set of samples was processed within 4 weeks and analyzed within 7 months. Fecal S100A12 concentrations were measured in 6 batches of all specimens using an established and validated species-specific in-house ^125^I-radioimmunoassay [26]. All samples were analyzed using the same batch of ^125^I-labelled S100A12 and other assay reagents.

### Data analysis

Assumptions of normal distribution and equal variances of numerical data were tested using a Shapiro-Wilk *W* and a Brown-Forsythe test, respectively. All summary statistics are reported as medians and interquartile ranges (IQR) or as percentages.

Statistical analyses were performed using non-parametric two-group (Wilcoxon rank-sum) or multiple-group comparisons (Kruskal-Wallis test), non-parametric correlation analysis (Spearman’s ρ correlation coefficient) and association testing between nominal variables (likelihood ratio or Fisher’s exact test, as appropriate).

Multivariate mixed (restricted maximum likelihood, or REML) models using log-transformed fecal S100A12 concentrations were constructed to evaluate the effect of different parameters (where *P* < 0.2 in univariate analyses) on fecal S100A12 concentrations. Sex, breed size, fecal score and the presence of enteropathogens were entered as fixed effects, whereas litter nested in kennel was considered as a random effect [24]. Statistical significance was set at a *P* < 0.05. Commercially available statistical software packages (JMP® v13.0, SAS Institute, Cary, NC, USA; Prism® v7.0, GraphPad Software, San Diego, CA, USA) were used for all statistical analyses.

## Results

### Study population

Puppies included in this study [median age (range): 7 (4−13) weeks; 152 females/126 males (sex not documented in 29 dogs)] were of 29 different breeds (all pure-bred dogs), the most common being Labrador Retrievers (*n* = 57) and German Shepherd dogs (*n* = 41). There were 78 small breed dogs (25%) and 229 large breed dogs (75%). Fecal scores ranged from 1–12 (median: 8, IQR: 6–9), with small breed dogs having significantly higher fecal scores (median: 9, IQR: 7–10) compared to large breed dogs (median: 7, IQR: 6–8; Wilcoxon rank-sum test: *Z* = 3.641, *P* = 0.0003).

### Study prevalence of selected endoparasites and viral agents

Positivity for tested gastrointestinal parasites ranged between 21 and 41%, whereas the study prevalence for both viruses evaluated reached approximately 20% (Table 1).

**Table 1:** Study prevalence of the different opportunistic pathogens tested in this study

Enteropathogen	No. of puppies tested	No. of positive test results	Study prevalence (%)
*Cystoisospora* spp.	307	117	38
*C. canis*	117	33	28
*C. ohioensis*	117	79	68
*C. canis* and *I. ohioensis*	117	5	4
*Toxocara canis*	301	63	21
*Giardia* spp.	305	126	41
Canine coronavirus (CCV)	303	61	20
Canine parvovirus (CPV)	301	54	18

There was an association between an abnormal fecal score and the presence of *C. canis* (likelihood ratio test: *χ*^2^ = 5.78, *df* = 1, *P* = 0.0163) as well as an infection with CPV2 (likelihood ratio test: *χ*^2^ = 26.36, *df* = 1, *P* < 0.0001). The presence of *C. canis* was associated with breed size and CPV2 infection (likelihood ratio test: *χ*^2^ = 16.16, *df* = 1, *P* < 0.0001 and likelihood ratio test: *χ*^2^ = 29.46, *df* = 1, *P* < 0.0001).

### Fecal S100A12 concentrations

Fecal S100A12 concentrations ranged from < 24−14,363 ng/g (median: 24 ng/g, IQR: 24−121 ng/g), with 20/307 dogs (6%) having a fecal S100A12 concentration above the upper limit of the reference interval (i.e. > 745 ng/g).

**Fig. 1:**
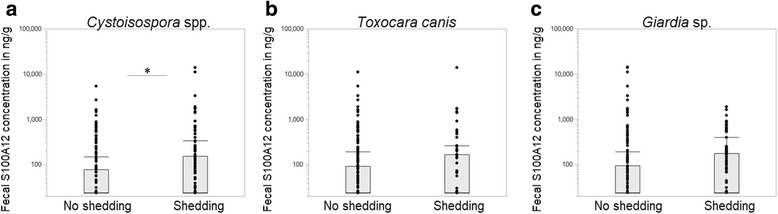
Univariate analysis of fecal S100A12 concentrations and endoparasite shedding in apparently healthy puppies (*n* = 307). **a** Dogs shedding *Cystoisospora* spp. had significantly higher fecal S100A12 concentrations than dogs not infected with these organisms (Wilcoxon rank-sum test: *Z* = 2.070, *P* = 0.0384). No significant difference in fecal S100A12 concentrations was seen between dogs shedding **b** *T. canis* (Wilcoxon rank-sum test: *Z* = 0.922, *P* = 0.3565) or **c** *Giardia* spp. (Wilcoxon rank-sum test: *Z* = 0.605, *P* = 0.5450) and those dogs from which these enteropathogens were not isolated. Boxes: interquartile range (IQR); vertical lines within boxes: medians; whiskers: determined by the outermost data points or values computed as (25th quartile: -1.5 × IQR) or (75th quartile: +1.5 × IQR). *Significant difference at *P* < 0.05; ***significant difference at *P* < 0.0001

**Fig. 2:**
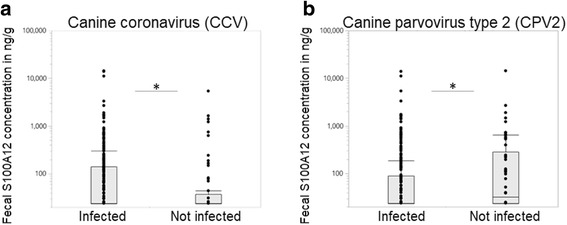
Univariate analysis of fecal S100A12 concentrations and the detection of viral agents in apparently healthy puppies (*n* = 307). **a** Dogs infected with CPV2 had significantly higher fecal S100A12 concentrations than dogs not infected with these organisms (Wilcoxon rank-sum test: *Z* = 2.202, *P* = 0.0277). **b** An infection with CCV produced significantly lower fecal S100A12 concentrations compared to dogs from which these viral agents were not isolated (Wilcoxon rank-sum test: *Z* = -2.115, *P* = 0.0345). Boxes: interquartile range (IQR); vertical lines within boxes: medians; whiskers: determined by the outermost data points or values computed as (25th quartile: -1.5 × IQR) or (75th quartile: +1.5 × IQR). *Significant difference at *P* < 0.05; ***significant difference at *P* < 0.0001

Univariate analysis revealed significantly higher fecal S100A12 concentrations in dogs infected with *Cystoisospora* spp. (Wilcoxon rank-sum test: *Z* = 2.070, *P* = 0.0384). However, there was no significant difference between those dogs infected or not with either *C. canis* (Wilcoxon rank-sum test: *Z* = 1.382, *P* = 0.1668) or *C. ohioensis*-complex (Wilcoxon rank-sum test: *Z* = 1.082, *P* = 0.2790) (Fig. 1, Table 2). Neither the presence of *T. canis* (likelihood ratio test: *χ*^2^ = 1.60, *df* = 1, *P* = 0.2064) nor *Giardia* spp. (likelihood ratio test: *χ*^2^ = 0.36, *df* = 1, *P* = 0.5498) was significantly associated with increased fecal S100A12 concentrations (Fig. 1, Table 2). Also, the numbers of *C. canis* (ρ_(301)_ = 0.08; *P* = 0.1956), *C. ohioensis* (ρ_(301)_ = 0.08; *P* = 0.1721) and *T. canis* (ρ_(301)_ = 0.06; *P* = 0.3451) did not correlate with fecal S100A12 concentrations. Fecal S100A12 concentrations were affected by CCV (Wilcoxon rank-sum test: *Z* = -2.115, *P* = 0.0345) or CPV2 infection (Wilcoxon rank-sum test: *Z* = 2.202, *P* = 0.0277) in univariate analysis (Fig. 2, Table 2).

**Fig. 3:**
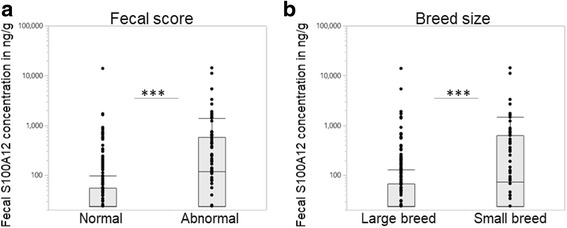
Univariate analysis of fecal S100A12 concentrations and the influence of fecal score and breed size in apparently healthy puppies (*n* = 307). **a** Dogs with an abnormal fecal score had significantly higher fecal S100A12 concentrations than dogs with a normal fecal score (Wilcoxon rank-sum test: *Z* = 5.749, *P* < 0.0001). **b** Small breed dogs had significantly higher fecal S100A12 concentrations than dogs of large breed size (Wilcoxon rank-sum test: *Z* = 4.879, *P* < 0.0001). Boxes: interquartile range (IQR); vertical lines within boxes: medians; whiskers: determined by the outermost data points or values computed as (25th quartile: -1.5 × IQR) or (75th quartile: +1.5×IQR). *Significant difference at *P* < 0.05; ***significant difference at *P* < 0.0001

**Table 2:** Evaluation of patient characteristics, opportunistic pathogen shedding, and fecal S100A12 concentrations in dogs included in this study (*n* = 307). *P*-values in bold font indicate a significant effect (*P* < 0.05)

Patient parameter or enteropathogen status	*n*	Fecal S100A12 concentration, median (IQR) (ng/g)	Fecal S100A12 above RI, *n* (%)	*P* (univariate analysis)	*P* (multivariate analysis)			
					Individual pathogens^d^	Individual pathogens^e^	Groups of pathogens	All entero-pathogens
Fecal score interpretation								
Abnormal^a^	75	118 (24–579)	14 (19)	**< 0.0001** (*Z* = 5.749)	**< 0.0001** (*F*_(1, 251)_ = 18.832)	**< 0.0001** (*F*_(1, 250)_ = 17.897)	**< 0.0001** (*F*_(1, 268)_ = 23.418)	**< 0.0001** (*F*_(1, 267)_ = 22.690)
Normal	223	24 (24–56)	6 (3)					
Age								
4–6 weeks	142	24 (24–136)	8 (6)	0.5025 (*χ*^2^ = 1.377, *df* = 2)	ns	ns	ns	ns
7–9 weeks	138	24 (24–95)	11 (8)					
≥10 weeks	27	24 (24–44)	1 (4)					
Sex								
Female	152	24 (24–150)	15 (10)	0.1670 (*Z* = -1.382)	0.5056 (*F*_(1, 234_ = 0.445)	0.4730 (*F*_(1, 231)_ = 0.517)	0.3683 (*F*_(1, 239)_ = 0.812)	0.4047 (*F*_(1, 243)_ = 0.697)
Male	126	24 (24–95)	5 (4)					
Breed size								
Small breed^b^	78	74 (24–633)	16 (21)	**< 0.0001** (*Z* = 4.879)	**0.0013** (*F*_(1, 52)_ = 11.562)	**0.0012** (*F*_(1, 53)_ = 11.733)	**0.0019** (*F*_(1, 53)_ = 11.655)	**0.0007** (*F*_(1, 52)_ = 13.127)
Large breed^c^	229	24 (24–67)	7 (3)					
*Toxocara canis*								
Shedding	63	24 (24–168)	6 (10)	0.3565 (*Z* = 0.922)	ns	ns	–	–
No shedding	238	24 (24–91)	12 (5)					
*Cystoisospora* spp.								
Shedding	117	24 (24–153)	9 (8)	**0.0384** (*Z* = 2.070)	0.2014 (*F*_(1, 96)_ = 1.655)	–	–	–
No shedding	184	24 (24–78)	9 (5)					
*Cystoisospora canis*								
Shedding	38	24 (24–223)	2 (5)	0.1668 (*Z* = 1.382)	–	0.1288 (*F*_(1, 90)_ = 2.350)	–	–
No shedding	263	24 (24–86)	16 (6)					
*Cystoisospora ohioensis*								
Shedding	84	24 (24–112)	8 (10)	0.2790 (*Z* = 1.083)	–	ns	–	–
No shedding	217	24 (24–101)	10 (5)					
*Giardia* spp.								
Shedding	126	24 (24–176)	7 (6)	0.5450 (*Z* = 0.605)	ns	ns	–	–
No shedding	179	24 (24–94)	13 (7)					
At least 1 parasite								
Shedding	222	24 (24–126)	14 (6)	0.3686 (*Z* = -0.899)	–	–	ns	–
No shedding	85	24 (24–87)	6 (7)					
CCV								
Infected	61	24 (24–38)	5 (8)	**0.0345** (*Z* = -2.115)	0.4505 (*F*_(1, 63)_ = 0.577)	0.4935 (*F*_(1, 66)_ = 0.474)	–	–
Not infected	242	24 (24–141)	15 (6)					
CPV2								
Infected	54	32 (24–283)	5 (9)	**0.0277** (*Z* = 2.202)	0.8138 (*F*_(1, 77)_ = 0.056)	0.8870 (*F*_(1, 77)_ = 0.020)	–	–
Not infected	247	24 (24–90)	15 (6)					
At least 1 virus								
Infected	114	24 (24–120)	10 (9)	0.9090 (*Z* = 0.114)	–	–	ns	–
Not infected	188	24 (24–127)	10 (5)					
At least 1 enteropathogen								
Shedding/infected	237	24 (24–138)	19 (8)	0.0881 (*Z* = -1.705)	–	–	–	0.2398 (*F*_(1, 86)_ = 1.401)
No shedding/not infected	70	24 (24–60)	1 (1)					

*Abbreviations*: *RI* reference interval, *ns* not significant

^a^Large breed puppies: ≤ 5, small breed puppies: ≤ 6 (4−5 weeks old) or ≤ 7 (≥ 6 weeks old) [25]

^b^Average adult weight: ≤ 25 kg [24]

^c^Average adult weight: > 25 kg [24]

^d^All *Cystoisospora* spp. grouped together

^e^*C. canis* and *C. ohioensis* considered separately

Age and sex were not found to affect fecal S100A12 concentrations (Kruskal-Wallis test: *χ*^2^ = 1.377, *df* = 2, *P* = 0.5025 and Wilcoxon rank-sum test: *Z* = -1.382, *P* = 0.1670, respectively) (Table 2), but the semiquantitative fecal score and fecal S100A12 concentration were inversely correlated (ρ_(298)_ = -0.28, 95% CI: -0.35– -0.12; *P* < 0.001). Breed size was also significantly associated with fecal S100A12 concentrations (Wilcoxon rank-sum test: *Z* = 4.879, *P* < 0.0001) (Fig. 3, Table 2).

On multivariate analyses, only the effect of fecal score (*F*_(1, 268)_ = 23.418, *P* < 0.0001) and breed size (*F*_(1, 53)_ = 11.655, *P* < 0.0019) remained significant (Table 2). Significance remained only for breed size (*F*_(1, 51)_ = 14.54, *P* = 0.0004) when evaluating the same parameters in multivariate models not including the parameter fecal score.

## Discussion

To the authors’ knowledge, the present study is the first to evaluate fecal S100A12 concentrations in apparently healthy dogs shedding enteropathogens (i.e. parasites, viral agents, or both). These preliminary data are important for further studies evaluating fecal S100A12 concentrations in dogs with chronic gastrointestinal diseases and are also of comparative relevance given the limitations of studying the effects of similar enteropathogens on fecal S100A12 concentrations in humans.

This study showed that fecal S100A12 concentrations in puppies are not affected by an infection with *Toxocara canis* or *Giardia* sp. In contrast, the presence of the parasite genera *Cystoisospora* spp. - independent of their number or subspecies - as well as an infection with the viral pathogens CCV or CPV2 were shown to be associated with alterations in fecal S100A12 concentrations on univariate analysis but not in a multivariate model. Thus, infections with enteropathogens evaluated in this study are not primarily associated with alterations in fecal S100A12 concentrations, whereas the concentration of fecal S100A12 is affected by the fecal score which reflects the overall health of the digestive tract. This is important for further studies evaluating fecal S100A12 concentrations in patients with suspected chronic gastrointestinal inflammation [27]. These findings are consistent with the results of a previous investigation in asymptomatic children showing fecal S100A12 concentrations to only have a tendency to be increased with concurrent *Giardia duodenalis* and helminth infections [20]. Also, the concentrations of fecal S100A12 measured in the study by Garzón et al. [20] were comparable to those concentrations detected in our study. Our results further agree with a previous study in adult people revealing no differences in fecal S100A12 concentrations in patients with viral gastroenteritis caused by Norwalk-like virus or rotavirus [17].

No difference in fecal S100A12 concentrations were detected between puppies testing positive for enteropathogens (except for *Cystoisospora* spp., CCV and CPV2 in univariate analysis) and those dogs testing negative in this study. This was still an unexpected finding given that S100A12 has been shown to have filariostatic or filaricidal properties (anti-parasite mechanism) through its binding to parasite paramyosin [10, 28] and the fact that S100A12 can be expressed in eosinophils in humans [29]. However, though endoparasites are more likely to be associated with eosinophilic or mixed inflammation [30], whether S100A12 expression in eosinophils is also induced or up-regulated in the context of inflammation in dogs has not been investigated. Also, experimental infection in cattle with *Echinococcus granulosus* was associated with a strong S100A12 response [31]. However, infection with *E. granulosus* results in a more granulomatous type of inflammation although other cells were also shown to express S100A12 at the site of infection-associated inflammation. These findings further suggest that limiting the availability of transition metal ions (i.e. Ca^2+^, Zn^2+^ and Mn^2+^) is not the mechanism of action to combat parasite or viral infections as opposed to infections with bacterial pathogens [32] or at least that S100A12 may not play a significant role in it. Though not demonstrated for S100A12, antimicrobial activity against *E. coli* has been shown for canine calprotectin [33].

Correlation of fecal S100A12 concentrations with only the fecal score and breed size suggests that fecal S100A12 concentrations are not primarily influenced by any single endoparasite or viral pathogen that were evaluated in this study. However, diarrhea in puppies is often multifactorial [24] and other factors that can influence the fecal score (e.g. stress, diet change, or other environmental factors) may also affect fecal S100A12 concentrations in dogs. The effect of dietary factors (e.g. diet type, feeding practices, dietary supplements, amount of milk ingested by suckling) on fecal S100A12 concentrations could not be evaluated in this study due to the large variety of these factors and the fact that the quantity of milk ingested cannot be determined. However, the association between fecal score and fecal S100A12 concentrations could also reflect an infection with another enteropathogen or enteropathogens for which puppies in this study were not evaluated. Small breed size was another significant factor for having an increased fecal S100A12 concentration in this study. Contrary to this, large breed size has been previously shown to be a determinant of lower fecal consistency [25], and this variable had been taken into account when interpreting fecal scores (normal/abnormal) in this study. Thus, one possible explanation for small breed dogs having a higher frequency of abnormal stools in this study could be an undetected infectious cause or environmental factors. However, lack of an ability of the 13-point scoring system [25] to sufficiently differentiate fecal qualities may also explain this finding, though this scoring system has been validated for use in puppies and appears to be superior to the 5-point system [34] that is often used to differentiate fecal qualities. The relationship between fecal S100A12 concentrations, fecal score, and breed size as well as its clinical implication, and whether the current results also translate to those in adult dogs, warrants further research.

Age has previously been reported to have no effect on the fecal concentrations of S100A12 [26], but dogs in that former study were significantly older (0.8–11.1 years; median: 4 years) than the dogs included in the present study (4–13 weeks; median: 7 weeks). Hence, age was also evaluated as a potential influencing factor as an age-related effect has been demonstrated for fecal calprotectin concentrations (another S100/calgranulin protein complex) in dogs [24]. In contrast to fecal calprotectin, age did not have an effect on fecal S100A12 concentrations in dogs, similar to previous findings in older puppies and adult dogs [26]. The reason for this difference between fecal calprotectin and fecal S100A12 is unknown. Possible explanations could be that S100A12 leakage into the still maturing gastrointestinal tract is negligible and that S100A12 production may be more specific to cells of the inflammatory response compared to calprotectin. However, these hypotheses need to be further investigated.

A reference interval for fecal canine S100A12 concentrations has been previously established (≤ 745 ng/g) using samples from older puppies and adult dogs [26]. Using this reference interval in puppies appears to be reasonable given that 6% of the dogs in the present study had a fecal S100A12 concentration above the upper limit of the reference interval. This fraction is similar to the fraction of the reference population (5%) that would be expected to have a fecal S100A12 concentration above the upper limit of the reference interval established by using the central 95th percentile [35]. Also, the upper limit of the reference interval of 745 ng/g in dogs is almost identical to that recently reported for children (750 ng/g) [36], suggesting that fecal S100A12 concentrations in dogs are comparable to those in people. As opposed to rodents [21], the S100A12 protein is encoded in the canine genome [22]. Thus, diseases in dogs appear to be an ideal model - and one that is superior to rodent models - for studying S100A12 biology and the RAGE-S100A12 axis in humans. Hence, future studies into the function and diagnostic utility of the S100A12 protein, as well as the S100A12-RAGE axis in chronic inflammatory conditions and their proposed use as novel selective therapeutic targets, should benefit from the present and also future studies in dogs.

We acknowledge that this study has some limitations. First, the small sample size in the analyses of subgroups of dogs carries the potential for type II error. Secondly, spot fecal samples were used for the detection of enteropathogens and also for measuring fecal S100A12 concentrations, but the biological variation and distribution of these enteropathogens and the S100A12 protein in fecal specimens in healthy dogs and dogs with gastrointestinal disease has not yet been reported. Lastly, histopathologic evaluation of gastrointestinal mucosal biopsies to determine whether enteropathogen shedding, a decreased fecal score, or both are associated with any clinically relevant mucosal lesions (i.e. quality and severity of inflammation) was not performed in the dogs in this study.

## Conclusions

We conclude that shedding of the enteropathogens *Cystoisospora* spp., *Toxocara canis*, *Giardia* sp., CCV and CPV2 does not appear to primarily affect fecal S100A12 concentrations in dogs. Thus, the presence of any one of these endoparasites or viral agents appears to be an unlikely cause of altered fecal S100A12 concentrations, but the fecal score as an indicator of the overall digestive health is reflected by the fecal S100A12 concentration. These preliminary data are an important starting point for further studies evaluating fecal S100A12 concentrations in dogs or when using fecal S100A12 concentrations as a biomarker in patients with chronic idiopathic gastrointestinal inflammation. In addition, the dog could be considered as an alternative model for studying S100A12 biology in humans.

## Abbreviations

CCV: canine coronavirus
CPV2: canine parvovirus type 2
EN-RAGE: RAGE-binding protein
IBD: inflammatory bowel disease
IQR: interquartile range
MMPs: matrix metalloproteinases
NF-kB: nuclear factor kappa B
RAGE: receptor of advanced glycation end products
REML: restricted maximum likelihood
S100A12: S100A12 protein
